# Ancient Microbes from Halite Fluid Inclusions: Optimized Surface Sterilization and DNA Extraction

**DOI:** 10.1371/journal.pone.0020683

**Published:** 2011-06-09

**Authors:** Krithivasan Sankaranarayanan, Michael N. Timofeeff, Rita Spathis, Tim K. Lowenstein, J. Koji Lum

**Affiliations:** 1 Department of Biological Sciences, State University of New York at Binghamton, Binghamton, New York, United States of America; 2 Laboratory of Evolutionary Anthropology and Health, State University of New York at Binghamton, Binghamton, New York, United States of America; 3 Department of Geological Sciences and Environmental Studies, State University of New York at Binghamton, Binghamton, New York, United States of America; 4 Department of Anthropology, State University of New York at Binghamton, Binghamton, New York, United States of America; The Centre for Research and Technology, Hellas, Greece

## Abstract

Fluid inclusions in evaporite minerals (halite, gypsum, etc.) potentially preserve genetic records of microbial diversity and changing environmental conditions of Earth's hydrosphere for nearly one billion years. Here we describe a robust protocol for surface sterilization and retrieval of DNA from fluid inclusions in halite that, unlike previously published methods, guarantees removal of potentially contaminating surface-bound DNA. The protocol involves microscopic visualization of cell structures, deliberate surface contamination followed by surface sterilization with acid and bleach washes, and DNA extraction using Amicon centrifugal filters. Methods were verified on halite crystals of four different ages from Saline Valley, California (modern, 36 ka, 64 ka, and 150 ka), with retrieval of algal and *archaeal* DNA, and characterization of the algal community using ITS1 sequences. The protocol we developed opens up new avenues for study of ancient microbial ecosystems in fluid inclusions, understanding microbial evolution across geological time, and investigating the antiquity of life on earth and other parts of the solar system.

## Introduction

Salt deposits, formed by evaporative concentration of brines at the Earth's surface, can be buried in the subsurface in relatively unaltered condition for hundreds of millions of years [1]–[7]. Halite crystals from these deposits and the tiny droplets of water in them, called fluid inclusions, have been reported to contain viable prokaryotes that may have survived encapsulated in the Earth's subsurface for geological periods [8]. Fluid inclusions in buried halite are a dark, hypersaline, low oxygen environment, which make them ideal repositories for preserving microorganisms and biomaterials, such as DNA, because they are not likely to be strongly effected by radiation, oxidative damage and hydrolysis [9]–[11]. A major question is whether fluid inclusions contain preserved DNA representative of the microbial diversity that existed in the paleo-environments in which the halite originally formed. If so, retrieval and characterization of ancient DNA trapped in fluid inclusions would lead to a better understanding of ancient ecosystems in extreme environments, evolution of microbial communities over geological time, and long-term preservation of biomaterials. Two recent studies [12], [13] have used PCR and DNA sequencing to examine microbial diversity in ancient fluid inclusions at the molecular level.

Contamination by modern microbes is a major concern with studies involving geologically ancient samples [14], [15]. Surface contamination of halite samples with modern microbial DNA can arise during their retrieval, handling and storage. Current surface sterilization protocols, including washes with alcohol or strong acids and alkalis, render surface microbes non-viable and prove adequate for retrieval of live microbes [11], [16]. These protocols, however, have not been fully evaluated for their effectiveness in complete removal of contaminating surface DNA, which is crucial for PCR-based genetic diversity studies. One way to evaluate the effectiveness of surface sterilization is with intentional contamination of the sample surface with DNA from a known unrelated organism (‘spiking’), followed by screening for its presence after decontamination and DNA extraction [14], [15], [17], [18]. Here, we used sample spiking with various combinations of decontamination agents, including but not limited to those previously described, to test 11 sterilization protocols (Table 1) and their effectiveness in destroying DNA lodged on halite crystal surfaces. The high salinities, large sample volumes and low DNA concentrations associated with processing halite samples affect DNA retrieval and its further characterization with PCR. We evaluated 12 widely used protocols based on silica extraction [19], isopropanol precipitation [20], DNA binding resins [13], in addition to Amicon centrifugal filters for DNA retrieval efficiencies from halite samples. DNA retrieval efficiency was calculated using variances in DNA yields obtained from a NanoDrop 3300 Fluorospectrometer with PicoGreen.

**Table 1:** List of surface sterilization protocols evaluated and the rinse steps for each.

Protocol Name	NaOH 10N Halite Saturated	HCl 10N	Halite SaturatedNa_2_CO_3_	Halite Saturated 6% Sodium hypochlorite (Bleach)	Halite Saturated Brine	Ethanol	Reference
Al	**+**	**−**	**−**	**−**	**+**	**−**	This study
AlB	**+**	**−**	**−**	**+**	**+**	**−**	This study
AlAc	**+**	**+**	**+**	**−**	**+**	**−**	Modified from Vreeland et al. *2000* [16]
AlAcBl	**+**	**+**	**+**	**+**	**+**	**−**	This study
Ac	**−**	**+**	**+**	**−**	**+**	**−**	This study
AcBl	**−**	**+**	**+**	**+**	**+**	**−**	This study
Bl	**−**	**−**	**−**	**+**	**+**	**−**	Modified from Kemp and Smith *2005* [18]
Et	**−**	**−**	**−**	**−**	**−**	**+**	Fish et al. *2002* [12]
BlEt	**−**	**−**	**−**	**+**	**+**	**+**	This study
AcBlEt	**−**	**+**	**+**	**+**	**+**	**+**	This study
AcEt	**−**	**+**	**+**	**−**	**−**	**+**	This study

We developed a robust surface sterilization protocol involving a soak in hydrochloric acid (10N HCl), neutralization with sodium carbonate (Na_2_CO_3_) and a 6% sodium hypochlorite (NaOCl) soak, followed by four rinses in halite saturated brine. We also describe a novel extraction protocol utilizing Amicon centrifugal filters for simultaneously increasing DNA concentrations while reducing salt concentrations, with consistent and efficient retrieval of DNA from small halite crystal samples (0.2–0.3 g).

**Figure 1:**
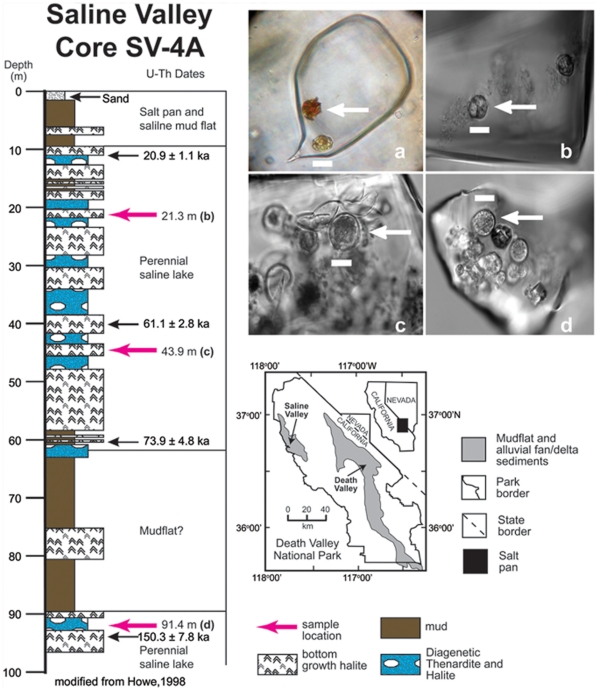
Map showing location of Saline Valley, California and stratigraphic column of core SV-4A, showing uranium series ages (black arrows), sediment types, paleoenvironments, and sample depths (red arrows). Modified from Howe (*1998*) and Lowenstein et al. (*2011*) [34]. Upper right shows fluid filled inclusions in halite crystals from Saline Valley with algal forms trapped inside (arrows). a) Two algal cells (probably *Dunaliella*) in fluid inclusion in modern halite collected in April 2004. The red color likely comes from *carotenoid* pigments found in the cells. b) Fluid inclusion in halite crystal from core SV-4A (21.3 m depth, ∼36 ka), with algal cells and clumps of smaller biomaterials, probably prokaryotes. c) Fluid inclusion in halite from core SV-4A (43.9 m depth, ∼64 ka). Arrow shows whole cell; above arrow, cells are in various stages of degradation including the glycocalyx (cell coat) of several ruptured algal cells. d) Fluid inclusion in halite from core SV-4A (91.4 m depth, ∼150 ka), showing many well-preserved algal cells. Scale bars are 10 microns in all images.

Our study of the DNA trapped in fluid inclusions in ancient halite involves a six-stage protocol. (1) First, crystals with microscopically identified cells and cell structures are selected (Figure 1) to increase the likelihood of DNA retrieval. (2) Next, human DNA is spiked onto the crystal surface. (3) Spiked samples are then surface sterilized with the acid-bleach ‘AcBl’ protocol and (4) DNA is extracted using the desalting protocol. Surface sterilization is verified by the lack of amplification using human mitochondrial HV1 primers. (5) PCR amplification is used for targeted retrieval of DNA from different microbial kingdoms. (6) PCR products are cloned and sequenced to characterize microbial diversity to the genus level.

## Results

### Surface Sterilization

**Figure 2:**
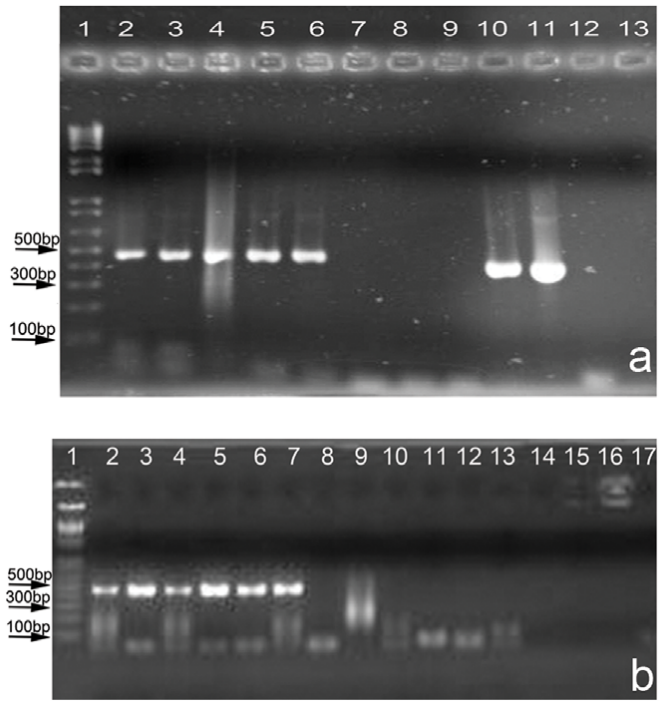
Agarose gel electrophoresis, Human HV1 PCR, Product size ∼440 bp. a) Evaluation of different surface sterilization protocols. Lane 1: DNA ladder, 2: Ac, 3:Al, 4:Bl, 5:AlBl, 6:AlAc, 7:AcBl, 8:AlAcBl, 9,12:Extract control, 10:Alcohol, 11:Spike, 13: PCR -ve. b) Effectiveness of surface sterilization. Halite crystals (modern Saline Valley) spiked with different amounts of human DNA. Lane 1: DNA ladder, Lanes 2,3,10,11: 1.5 ng spike, Lanes 4,5,12,13: 3.0 ng spike, Lanes 6,7,14,15: 4.5 ng spike, Lanes 8,9,16,17: no spike. Lanes 2–9: no surface sterilization. Lanes 10–17 surface sterilized.

The effectiveness of 11 surface sterilization protocols (listed in Table 1) on modern halite crystals (Saline Valley 2004) was evaluated by testing for residual spiked human DNA following surface sterilization, using PCR primers targeting the HV1 region [21] of the human mitochondrion. The results (Figure 2a) indicate that several existing protocols including Vreeland et al. *2000* [16] (Figure 2a, Lane 6), and Fish et al. *2002* [12] (Figure 2a, Lane 10) fail to completely remove spiked human DNA (presence of HV1 PCR products). Among the other protocols tested, the addition of a bleach wash (6% sodium hypochlorite) to the Vreeland et al. *2000* [16] protocol (alkali-acid-bleach, “AlAcBl” on Table 1), or the use of an acid and bleach wash (“AcBl” on Table 1) proved sufficient in removing contaminating DNA (lack of HV1 PCR products, Figure 2a, Lanes 7, 8). A bleach wash was required to completely destroy surface DNA.

#### Effectiveness of Surface sterilization

**Figure 3:**
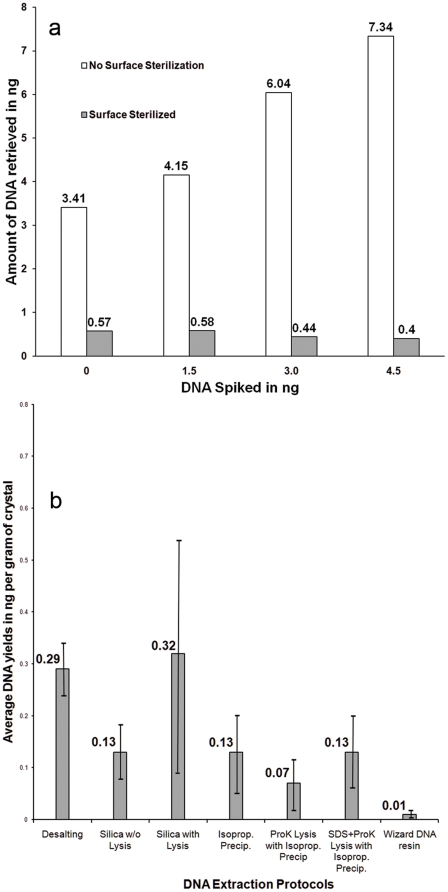
DNA quantification using PicoGreen®. a) Effectiveness of surface sterilization: average DNA yields, in ng, before and after surface sterilization for different spike amounts. b) Plot of average DNA yields in ng per gm of crystal for different extraction protocols.

Sets of modern halite crystals from Saline Valley (collected in 2004) were spiked with known amounts of human DNA (1.5, 3.0, and 4.5 ng). Paired DNA extractions (using desalting) were performed on crystals for each spiked amount, with one subset being surface sterilized using the acid bleach “*AcBl*” protocol (Table 1) and the other subset non-surface sterilized prior to extraction. DNA yield was quantified for each of the three crystal spike trials (1.5, 3.0, and 4.5 ng), with and without surface sterilization (Figure 3a). DNA yield from non-surface sterilized crystals increases proportionally to amount of DNA spiked. DNA yields from surface-sterilized crystals however, are relatively constant and independent of the amount of spike DNA used. Further, an ∼80% decrease in DNA yield was observed before and after surface sterilization from unspiked crystals.

PCR assays with human HV1 primers showed complete removal of spiked DNA from the surface sterilized crystal subset (Figure 2b). In addition, a second round of PCR (‘booster’ PCR) [22], was performed with 2 µl of product from the first PCR as template, assuming amplification below visual detection. No amplification was detected from the surface sterilized crystals following booster PCR, confirming the absence of even trace amounts of spiked human DNA.

### DNA extraction

DNA was extracted from modern halite crystals (Saline Valley 2004) using 12 different protocols following surface sterilization by the acid-bleach method (Table 1, *AcBl*). DNA yields were quantified using the NanoDrop 3300 Fluorospectrometer. Average DNA yields, normalized per gram of crystal for the various extraction protocols (Figure 3b), show that the desalting protocol using Amicon centrifugal filters, followed by a Qiagen spin column purification, gave the best results (average yields of 0.29 ng and ∼17% variance). Silica extractions with lysis yield similar average DNA (0.32 ng) but larger variance (∼69%). Other protocols, including silica without lysis and isopropanol-based extractions, have lower yields (0.07 ng–0.13 ng) with larger variances ranging from ∼30–70%. Extracts from the Wizard® DNA protocol had average yields less than 0.01 ng.

### Algal Diversity in Ancient Crystals

**Figure 4:**
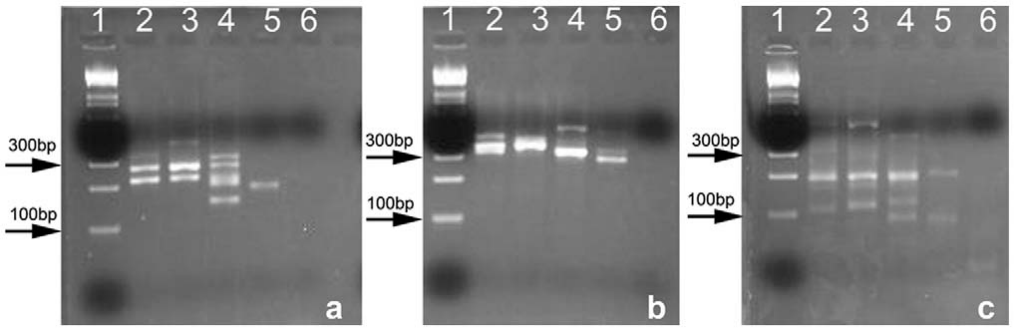
Agarose gel electrophoresis images from a) Algal ITS1 (∼100–300 bp), b) Algal ITS2 (∼300 bp) and c) *Archaeal* 16s (∼200 bp). Sample order: Lane 1: DNA ladder, 2: Modern Saline Valley, 3: Saline Valley ∼36 ka, 4: Saline Valley ∼64 ka, 5: Saline Valley ∼150 ka, and 6: PCR –ve.

Modern and ancient halite crystals (36 ka, 64 ka, 150 ka, Figure 1) from Saline Valley were surface sterilized using the acid bleach *AcBl* protocol and DNA was extracted using the desalting protocol. The DNA extracts were screened using PCR with primers specific to Algal ITS1, ITS2 [23] and *Archaeal* 16s rDNA [24], [25]. All four samples showed positive amplification with expected fragment sizes for the primers used (Figure 4).

**Figure 5:**
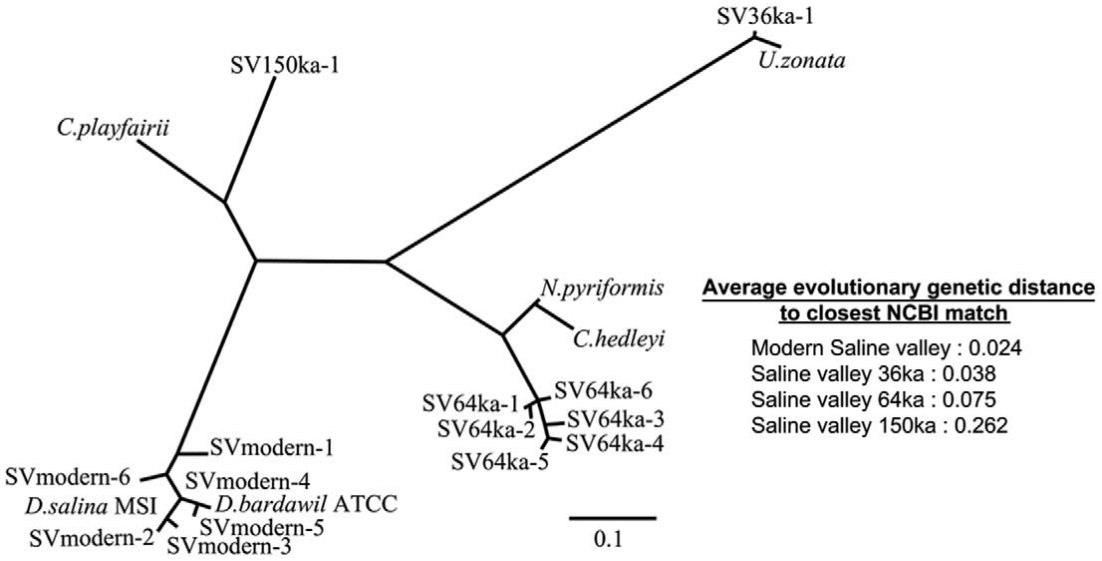
Unrooted Maximum likelihood tree, Saline Valley ITS1 algal sequences and closest matches from extant sequences deposited in the NCBI database. Distinct clusters are observed for the 4 different time periods. Bottom right shows average evolutionary distance between sequences and their closest NCBI match.

ITS1 PCR products were cloned and sequenced to obtain preliminary estimates of algal diversity. Fourteen unique algal ITS1 sequences (GenBank accession: HQ386893–HQ386906) were identified from a total of 83 algal ITS1 fragments retrieved across the four time periods. Sequence analysis using BLAST [26] searches allowed for genus level identification of *Dunaliella* in modern halite and *Ulothrix* in 36 ka halite. Sequences from the 64 ka and 150 ka samples were identified as belonging to green algae, with closest matches to *Nephreselmis* (∼80% sequence similarity) and *Chloromonas* (∼83% sequence similarity) respectively. An unrooted Maximum likelihood tree was generated using ClustalW [27], PhyML [28] and *drawtree* [29], for the 14 unique algal ITS1 fragments and their closest matches from the NCBI database (Figure 5). Average evolutionary distances between retrieved sequences and closest database matches were calculated from the phylogenetic tree (Figure 5). Algal diversity within the four samples, as measured by their heterozygosities [30], appears to be independent of sample age. The 36 ka and 150 ka samples have a heterozygosity of zero. The 64 ka samples have a heterozygosity of 0.34, similar to modern samples (0.38), despite a ∼40% decrease in number of sequences.

## Discussion

We identified two protocols (acid-bleach, *AcBl*, and alkali-acid-bleach, *AlAcBl*, Table 1) that consistently removed contaminating surface DNA. The *AcBl* protocol was chosen for surface sterilization because it requires less sample manipulation. The increase of retrieved DNA proportional with spike amount (Figure 3a, no-surface sterilization series) demonstrates the capacity of contaminating DNA to adhere to halite crystal surfaces.

Particular care should be taken to ensure complete neutralization of HCl following the acid wash. Residual acid adversely affects DNA extraction by degrading the Millipore filter membranes, and also results in hydrolysis of DNA in the final extract. Bleach carryover also causes DNA degradation and inhibition of PCR, hence complete removal of bleach via multiple washes in halite saturated brine is imperative.

Conventional DNA extraction protocols such as silica extraction, alcohol precipitation, DNA binding resins etc., often involve selective binding or separation of DNA based on specific buffer conditions. The high variances observed in DNA yields for these protocols can be potentially attributed to the large starting volumes and low sample DNA concentrations. In addition to inconsistent yields, the protocols employing the above reagents also show variation in PCR (results not shown), likely due to carryover of silica, Wizard resin particles, and incomplete removal of salt.

The Amicon centrifugal filters retain only molecules larger than 50 Kilodalton (KDa) and DNA fragments longer than 50 bp. Further, the filters were specifically designed to function in high salt conditions common in buffer exchanges and sample concentration protocols. The Amicon filter columns provide a >10 fold increase in DNA concentration, along with a decrease in salt concentration from ∼1 M to <20 µM. This allows for improved DNA retrieval following purification with the Qiagen spin columns. We therefore judge this desalting protocol to be the most effective when extracting small amounts of DNA from fluid inclusions in halite.

Preliminary sequencing of ITS1 PCR products from modern surface halite and Saline Valley core samples shows algal diversity changes with time. Microscopic analysis (Figure 1) of halite crystals shows algal cells within fluid inclusions that are similar in morphology and size to the common halophilic alga *Dunaliella salina* [31], [32]. DNA from fluid inclusions in modern surface halite was identified as derived from *Dunaliella*. DNA sequence analyses from ancient fluid inclusions, however, show non-overlapping genera of algae for each of the 3 ancient samples (Figure 5, 36 ka, 64 ka, 150 ka). Although the ancient algae visualized in fluid inclusions resemble *Dunaliella*, the DNA sequences from these ancient samples suggest a change in algal community composition over time. The phylogenetic tree and derived evolutionary distances (Figure 5) show a progressive increase with age between retrieved sequences and their closest matches among extant organisms deposited in the NCBI database. This could potentially be an effect of evolutionary divergence, changing abundances and paleoenvironments over time, extinctions, or a limitation of the database queried. Persistence of modern contaminating DNA following surface sterilization is expected to result in retrieval of identical sequences from all the samples. Thus, the non-overlapping diversity from the four Saline Valley samples is consistent with effective surface sterilization.

Studies aimed at characterizing the microbial community structure in fluid inclusions in ancient halite will prove valuable in reconstructing the ecosystems in which they originally lived, understanding the microenvironments in which they were confined, including the conditions that may have allowed some prokaryotes to survive within fluid inclusions for extended periods. Samples of halite from the subsurface of Saline Valley (0–150 ka), Death Valley (0–200 ka) and Searles Lake (0–2 Ma), California, will allow study of the evolution of microbial communities in localized geographical space (arid closed basins) across geological time, which will provide insights into the effect of geological processes on the evolution and distribution of biological communities. Older halites, up to hundreds of millions of years in age, such as the Salado salt of New Mexico (250 Ma) [2], [6], [16], [33], the Wellington Formation, Kansas (277–283 Ma) [33], and the Silurian Salina Salt, Michigan (416–418 Ma) [7], [13], allow study of the preservation of DNA in deep geological time, investigate the evolution of microbial life on earth, and the influence of climate change on biological diversity and community composition.

## Materials and Methods

### Materials

Halite crystals were obtained from Saline Valley, California (Figure 1). Modern halite crystals were sampled from current surface deposits, collected in 2004 and 2007. Older halite samples were obtained from core SV-4A, at depths of 21.3 m (∼36 ka), 43.9 m (∼64 ka), and 91.5 m (150 ka) [34]. Crystals were microscopically examined (Zeiss AXIO Imager.A1, Plan Apochromatic 100×/1.4 NA oil objective, Zeiss AxioCam MRm for B&W images and Nikon Coolpix 8800 for color images) to verify the presence of microbial cells or cellular structures, including probable halophilic algae and prokaryotes, within fluid inclusions [31], [32], [34], [35] (Figure 1).

All solutions used for surface sterilization were prepared at halite saturation to minimize crystal dissolution with the exception of 10N HCl (minimal halite solubility). Halite saturated NaCl and Na_2_CO_3_ solutions, used in washes and acid neutralization respectively, were freshly prepared, autoclaved (121°C, 15 psi for 20 minutes) and UV sterilized for 60 minutes. Nanopure water (Millipore filtered+UV sterilized), used in extractions, was autoclaved (121°C, 15 psi for 20 minutes) and UV sterilized for 3 hours. Fresh aliquots of reagents were prepared prior to use in surface sterilization and extraction experiments.

Surface sterilization and DNA extraction were performed under a Class IIA laminar flow hood in a dedicated ancient DNA facility. PCR reactions were set up in a separate room in the ancient DNA facility. All working surfaces were sterilized with bleach, and UV treated for 3 hours before and after use.

### Surface Sterilization

Samples were spiked with known quantities of human DNA extract mixed with bromophenol blue dye (0.05% w/v). The dye provides visual verification of intentional surface contamination, and helps visualize imperfections and microfractures on the crystal surface. Further, the dye is an acid-base indicator, allowing verification of acid-neutralization.

Effectiveness of various combinations of sterilization agents such as 10N HCl, 10N NaOH, 6% sodium hypochlorite (bleach) and 100% ethanol in removal of spiked human DNA was evaluated, including previously published protocols (Table 1). Acid, alkali, and bleach washes were performed by soaking crystals for 15 minutes in 4 ml of 10N HCl, 10N halite saturated NaOH and halite saturated bleach, respectively. Acid was neutralized by soaking crystals in 4 ml of halite saturated Na_2_CO_3_ for 15 minutes. Crystals were soaked in 4 ml halite saturated brine (15 minutes) between each sterilization stage to minimize reagent carryover. A final set of four washes in 4 ml of halite saturated brine was performed prior to DNA extraction.

### DNA Extraction

Various protocols were evaluated for their efficiency in DNA retrieval from ∼0.2 g of modern halite crystals (Saline Valley 2004). Protocols tested include variations of previously published methods such as silica extraction [19], alcohol precipitation [20], use of DNA binding resins such as the Wizard® DNA Clean-Up System from Promega [13], and size exclusion filtration (Amicon Ultra-0.5 50 kDa, Millipore). Further, the effect of various lysing agents such as SDS (sodium dodecylsulphate), proteinase K, and L6 buffer [19] on extraction yield and variance was evaluated. Extractions were performed in triplicate to calculate yield consistency.

#### Silica extraction

Crystals (∼0.2 g) were dissolved in 600 µl of L2 (denaturation) [19] or L6 (lysis) buffers (3 hrs incubation at 56°C). Sixty µl of equilibrated silica solution was added and samples incubated with mixing for 10 minutes at 56°C. The samples were then centrifuged at 20,000 rcf (relative centrifugal force) for 20 seconds and the supernatant was discarded without disturbing the silica pellet. The pellets were washed twice in 300 µl of L2 buffer, followed by two washes in 70% ethanol and allowed to dry. Pellets were resuspended in 100 µl TE buffer by incubating and mixing for 15 minutes at ∼50°C. Finally, the solution was centrifuged at 20,000 rcf for 20 seconds and the supernatant containing DNA in TE was pipetted out and stored at 4°C for quantification and PCR.

#### Alcohol precipitation

Sets of alcohol precipitation experiments were performed with and without various lysing agents (proteinase K, SDS, SDS+proteinase K, and no lysis). Crystals (∼0.2 g) were dissolved completely in 700 µl nanopure water to yield a final salt concentration of ∼5 M. Forty µl proteinase K (20 mg/ml) and/or SDS (0.5% final concentration) were added to the mixture and samples incubated at 56°C for 4 hrs, followed by addition of 2–3 volumes of 80% isopropanol. In the no-lysis extraction, 2–3 volumes of 80% isopropanol were added directly to the dissolved sample. The samples were then mixed by inverting the tubes and incubated overnight at 4°C. The samples were then centrifuged at 20,000 rcf for 1 hour and the supernatant discarded. Finally, the pellet was washed with 70% ethanol to remove any coprecipitated impurities, dried, and resuspended in 70 µl TE buffer.

#### Wizard DNA resin purification

Crystals (∼0.2 g) were dissolved in 700 µl nanopure water with the addition of various combinations of lysing and denaturing agents including proteinase K, SDS, L2, and L6 buffers [19]. Three hundred µl of the Wizard® DNA binding resin was added to the samples and mixed. The DNA was then purified following the manufacturers protocol and eluted in 70 µl TE buffer.

#### Desalting

Crystals (∼0.2 g) were dissolved in 3.5 ml nanopure water to a final salt concentration of 1 M and centrifuged at 3,000 rcf for 8 minutes in Amicon centrifugal filters (Ultra-0.5 50 kDa,YM-50, Millipore). The size-exclusion filters retain DNA fragments longer than 50 bp and simultaneously concentrate the sample while removing the salt. Following each round of centrifugation, nanopure water was added to bring filtrate volume to 500 µl. Following 5–6 rounds of centrifugation, 100 µl of nanopure water was added to the column and the DNA was eluted by centrifuging upside-down at 4,000 rcf for 6 minutes. The raw extract was then purified using Qiagen spin columns to remove any humic substances, secondary metabolites and other potential PCR inhibitors.

### DNA quantification

DNA was quantified on a NanoDrop 3300 Fluorospectrometer using PicoGreen® (Quant-iT™ PicoGreen ® dsDNA kit, Invitrogen) following the manufacturer's protocol. DNA detection sensitivity for the Nanodrop 3300 with PicoGreen® is ∼20 pg/µl. Modern halite DNA extracts from the Wizard® DNA protocol and ancient extracts (64 ka, 150 ka) were below the detection limit of the instrument, and requantification was attempted following a second round of concentration with a final elution volume of ∼5 µl.

### PCR

PCR reactions were carried out in 12.5 µl volumes containing 1× PCR buffer (Invitrogen), 200 µM dNTPs each, 1.5 mM MgCl_2_, and 0.5 µM primers. Two to three µl of DNA template was added to each reaction. Primers H15996 and L16401.1 (modified from Vigilant et al. *1990*) [21], amplifying a ∼440 bp segment of the human mitochondrial genome (HV1 region) were used to assay for the presence of spiked DNA following surface sterilization. Universal primers A344f (5′ ACGGGGCGCAGCAGGCGCGA 3′) [24] and A518r (5′ ATTACCGCGGCTGCTGG 3′) [25] targeting a 200 bp fragment of 16s rDNA were used to screen for the presence of Archaeal and Bacterial DNA. Primers ITS1(5′ TCCGTAGGTGAACCTGCGG 3′), ITS2 (5′ GCTGCGTTCTTCATCGATGC 3′) targeting the ribosomal ITS1 region and primers ITS3 (5′ GCATCGATGAAGAACGCAGC 3′) and ITS4 (5′ TCCTCCGCTTATTGATATGC 3′) were used to screen for algal DNA [23]. PCR reactions were carried out on a GeneAmp® PCR System 9700 from Applied Biosystems. The primers used were tested at multiple annealing temperatures, ranging from 60°C to 45°C, and were found to optimally amplify their respective templates at an annealing temperature of 55°C. All PCRs were run with the following conditions: initial denaturation at 94°C, followed by 50 cycles of 30 sec at 94°C (denaturation), 30 sec at 55°C (annealing) and 30 sec at 72°C (extension), and a final extension of 10 mins at 72°C. A fifty cycle reaction is recommended to ensure successful amplification. All PCR reactions were replicated a minimum of four times to ensure consistency. Two ml aliquots of halite saturated NaCl, halite saturated Na_2_CO_3_ (surface sterilization solutions), and nanopure water (crystal dissolution) were extracted with each batch of crystals, and served as controls for the surface sterilization and extraction process. PCR for archaeal/bacterial, algal and human DNA fragments (primers specified above) was used to screen and verify sterility of control extracts. In addition, PCR reactions without addition of samples were included as negative controls to verify sterility of PCR reagents [14]. Products were visualized in a 1.2% agarose gel, stained with ethydium bromide.

### Cloning and DNA Sequencing

Purified ITS1 PCR products were cloned using Qiagen PCR cloning kits following the manufacturer's protocols. A subset of colonies per sample was purified and cycle sequenced using ABI's BigDye® Terminator v3.1 system, followed by sequencing on a 96 capillary ABI 3730xl genetic analyzer.

### Sequence analysis

Sequences were characterized to the genus level using BLAST [26] searches against the NCBI non-redundant nucleotide database (nr). Sequences were then aligned using ClustalW [27] and Maximum likelihood trees were generated using the PhyML package [28]. Trees were visualized using the *drawtree* program in PHYLIP [29]. Online versions of all three programs are available through the Phylogeny.fr portal (http://www.phylogeny.fr/version2_cgi/phylogeny.cgi) [36]. Heterozygosities were calculated using the equation below, where *‘h’* is the heterozygosity, *‘x_i_’* is frequency of the ith phylotype, *‘n’* is the sample size, and *‘k’* is the total number of sequences 
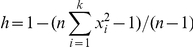
, derived from Nei and Roychoudhury, 1974 [30]. Retrieved sequences were deposited in NCBI with GenBank Accession codes HQ386893–HQ386906.
